# Dosimetric Feasibility of Utilizing the ViewRay Magnetic Resonance Guided Linac System for Image-guided Spine Stereotactic Body Radiation Therapy

**DOI:** 10.7759/cureus.6364

**Published:** 2019-12-12

**Authors:** Gage Redler, Tynan Stevens, Jochen Cammin, Martha Malin, Olga Green, Sasa Mutic, Sean Pitroda, Bulent Aydogan

**Affiliations:** 1 Radiation Oncology, Moffitt Cancer Center, Tampa, USA; 2 Medical Physics, Dalhousie University, Halifax, CAN; 3 Radiation Oncology, Washington University School of Medicine, Barnes-Jewish Hospital, St. Louis, USA; 4 Radiation Oncology, New York University, Langone Medical Center & Laura and Issac Perlmutter Cancer Center, New York, USA; 5 Radiation and Cellular Oncology, University of Chicago, Chicago, USA

**Keywords:** spine sbrt, igrt, mr-igrt, magnetic resonance-guided radiation therapy (mrgrt), dosimetry treatment planning

## Abstract

Introduction: Spine stereotactic body radiation therapy (SBRT) achieves favorable outcomes compared to conventional radiotherapy doses/fractionation. The spinal cord is the principal dose-limiting organ-at-risk (OAR), and safe treatment requires precise immobilization/localization. Therefore, image guidance is paramount to successful spine SBRT. Conventional X-ray imaging and alignment to surrogate bony anatomy may be inadequate, whereas magnetic resonance imaging (MRI) directly visualizes the dose-limiting cord. This work assessed the dosimetric capability of the ViewRay (ViewRay Inc. Oakwood Village, OH) magnetic resonance (MR) guided linac (MR-Linac) for spine SBRT.

Methods: Eight spine SBRT patients without orthopedic hardware who were previously treated on a TrueBeam using volumetric modulated arc therapy (VMAT) were re-planned using MR-Linac fixed-field intensity-modulated radiation therapy (IMRT). Phantom measurements using film, ionization chamber, and a commercial diode-array assessed feasibility. Plans included a variety of prescriptions (30-50 Gy in 3-10 fractions).

Results: MR-Linac plans satisfied all clinical goals. Compared to VMAT plans, both entrance dose and heterogeneity increased (D_max_: 134±3% vs. 120±2%, p=0.0270), while conformality decreased (conformity index: 1.28±0.06 vs. 1.06±0.06, p=0.0005), and heterogeneity increased. However, while not statistically significant, MR-linac cord sparing improved (cord D_max_: 16.1±2.7Gy vs. 19.5±1.6Gy, p=0.2066; cord planning organ at risk volume (cord PRV) D_max_: 20.0±2.6Gy vs. 24.5±2.0Gy, p=0.0996). Delivery time increased but was acceptable (14.39±1.26min vs. 9.57±1.19min). Ionization chamber measurements agreed with planned dose to within 2.5%. Film and diode measurements demonstrated accurate/precise delivery of dose gradients between the target and the cord.

Conclusion: Spine SBRT with the MR-Linac is feasible as verified via re-planning eight clinical cases followed by delivery verification in phantoms using film, diodes, and an ionization chamber. Real-time visualization of the dose-limiting cord during spine SBRT may enable cord-based gating, reduced margins, alternate dose schemas, and/or adaptive therapy.

## Introduction

Nearly 40% of cancer patients present with clinically symptomatic spinal disease and ~90% demonstrate evidence of disease upon autopsy [1]. Treatment options include systemic therapy, surgery, radiation therapy (RT), or surgery+RT, with goals of pain palliation, bone fracture avoidance, and/or management of neurological symptoms and disease progression [2]. In many settings, surgery+RT is standard of care. Ablative stereotactic body RT (SBRT) may provide improved efficacy compared to conventional fractionation [1]. Furthermore, spine SBRT has improved potential for retreatment and dose escalation for radioresistant disease.

Accuracy and precision are paramount to spine SBRT, thereby emphasizing patient immobilization and image guidance. The primary dose-limiting organ-at-risk (OAR) is the spinal cord. Cord definition is usually from magnetic resonance imaging (MRI) (or computed tomography (CT) myelography). Cord delineation uncertainty, inability to visualize/immobilize the cord during treatment, and the importance of respecting cord tolerances often lead to a cord planning organ-at-risk volume (PRV). Use of PRV margin, typically 1-2 mm, decreases disease coverage [1]. Prior to image-guided RT (IGRT), a framed approach provided immobilization for linac-based spine SBRT [3]. Initial IGRT attempts used a vacuum bag immobilization device and ultrasound [4]. Real-time image guidance (orthogonal kVs) with Cyberknife enabled frameless spine SBRT by providing setup uncertainties of 1.0-1.2 mm [5-6]. Similar approaches are now widely adopted using traditional linac approaches and kV image guidance (2D planar images and/or 3D cone beam CT (CBCT)).

An issue with conventional kV imaging is that positioning tends to rely on bony anatomy, which is a surrogate for the anatomy of interest (e.g., tumor or cord). MRI can address this and already has important roles in RT, specifically spine SBRT [1-2]. Factors limiting utilization of MRI in RT (e.g., logistics of having a dedicated MRI, geometric distortion, availability during treatment), have mostly been addressed with the advent of MR guided RT (MRgRT) [7-8]. One such implementation is the MRidian MR-linac (ViewRay Incorporated, Oakwood Village, OH) [7]. The utility of MRidian images has been compared to CT and on-board CBCT. No significant volumetric differences were found between contours drawn using CT versus ViewRay MRI [9]. Compared to on-board CBCT, MRIdian images were mostly superior, with the major shortcoming being bony anatomy visualization, which is less pertinent when soft tissue (e.g., cord) is directly identifiable [10].

The MRIdian MR-linac system (6MV flattening filter free) has improved dosimetric qualities compared to a prior cobalt-based system (e.g., higher multileaf collimator (MLC) resolution, improved penetration, decreased penumbra, faster treatment) [11-14]. Specifically, the MR-linac system’s MLCs have a width of 8.3 mm but are double-stacked and offset to provide a 4.15 mm effective leaf width, versus 10.5 mm for the cobalt system. MLCs for both systems are double-focused (see [15] for details on system technical specifications). One planning study with the ^60^Co MRIdian suggested that spine SBRT was not dosimetrically feasible [13]. MRgRT spine SBRT may be feasible with the MR-linac system, and may improve upon kV-based IGRT approaches. Preliminary data suggests that aligning to vertebra versus cord, which is visible with MRIdian, requires an average shift of 0.9 mm (up to 1.8 mm observed; no obvious time trends) [16].

This work demonstrates the dosimetric feasibility of spine SBRT with the ViewRay MR-Linac system, which may enable visualization of the dose limiting spinal cord during treatment and allow cord-based gated delivery and/or online adaptive spine SBRT. Eight spine SBRT plans (clinically planned using VMAT on a conventional linac) are compared to MR-Linac plans. Fidelity between planned and delivered MR-linac dose distributions is verified using phantom measurements with film, diodes, and ion chambers.

## Materials and methods

Clinical cases

**Figure 1:**
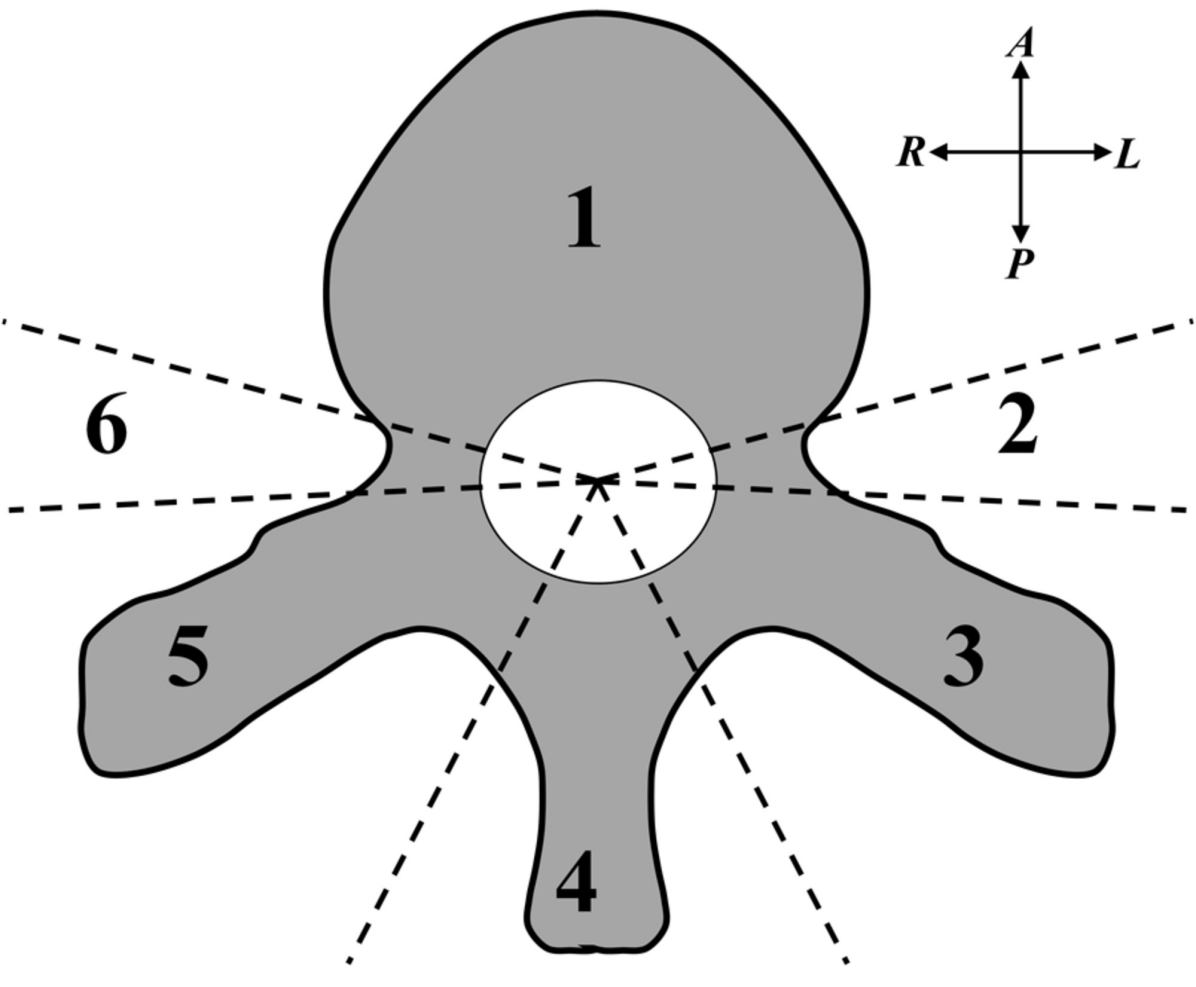
Spinal Vertebrae Diagram Diagram of spinal vertebrae (axial plane) showing different regions by which to describe the extent of the planning target volume (PTV) to be treated. 1 = vertebral body, 2 = left pedicle, 3 = left transverse process, 4 = spinous process, 5 = right transverse process, 6 = right pedicle. The spinal canal containing the spinal cord is shown as the central white oval area.

Eight spine SBRT plans recently treated in our clinic were re-planned in the ViewRay treatment planning system (TPS). Plans included prescription doses ranging from 30 to 50 Gy and fractionation schemes ranging between three and ten fractions. One plan incorporated a simultaneous integrated boost. PTV sizes ranged from 25-160 cc. PTV extent ranged from anterior vertebral body only, to various combinations of pedicle, transverse process and spinous process coverage (see Figure 1 for delineation of regions of potential disease extent; see Table 1 for corresponding descriptions of targets used in this work), based upon the international spine radiosurgery consortium target volume guidelines [2].

**Table 1:** Plan Characteristics Description of treatment plans included in this study. Plan #4 included a simultaneous integrated boost, which is indicated by the additional volume and Rx dose in parenthesis.

Case # (Vertebral Level)	PTV Volume [cc]	PTV Extent	Prescription Dose / Fractionation	PTV Clinical Coverage Goal(s)
1 (T)	60.2	1, 2, 6	30Gy / 3fx	V_100%_ > 95%
2 (T)	119.9	1, 2, 3, 5, 6	50Gy / 5fx	V_100%_ > 80% ; V_90%_ > 95%
3 (T)	38.2	1, 2, 4, 5, 6	30Gy / 3fx	V_100%_ > 95%
4 (L/S)	160.3 (9.1)	1, 2, 4, 5, 6	30(40)Gy / 5fx	V_100%_ > 95%
5 (T)	36.3	1, 2, 3, 4, 6	30Gy / 3fx	V_100%_ > 90% ; V_90%_ > 98%
6 (L)	73.7	1, 2, 6	30Gy / 3fx	V_100%_ > 90% ; V_90%_ > 98%
7 (L)	25.8	1	30Gy / 3fx	V_100%_ > 95%
8 (T)	71.2	1, 2, 4, 5, 6	30Gy / 3fx	V_100%_ > 90%

Planning approach

The principal dose-limiting OARs were the spinal cord and esophagus. Cord OAR definition was either based on CT myelogram (n=6) or substituted with spinal canal (n=2). Cord planning organ at risk volume (PRV) used a 1 mm expansion. When spinal canal was a cord surrogate, no PRV expansion was used. Some plans were treating regions in the lumbar/sacral vertebral level, where cord transitions to cauda equina, however, for generality, this distinction is not made (i.e., cauda = cord). Secondary OARs include esophagus, bowel, liver, and lungs. These were not dose-limiting and are not presented in the following analyses. OAR goals were from the American Association of Physicists in Medicine (AAPM) TG-101 [17]. All images and structures (targets, OARs, PRVs) were consistent between clinical VMAT plans and MR-Linac plans.

Conventional linac plans were VMAT and used a Varian TrueBeam accelerator with HDMLC and a 6MV beam. The plans used 2-4 co-planar arcs. PTV coverage goals were V_100%_ > 95% unless OAR constraint(s) precluded this, in which case either fractionation was reconsidered or lower PTV coverage was accepted. Eclipse v11 (n=7) (Varian Medical Systems, Inc., Palo Alto, CA) or Pinnacle v9 (n=1) (Philips Radiation Oncology Systems, Fitchburg, WI) were used. Dose was calculated with 1 mm resolution, using homogeneity correction (collapsed cone convolution superposition in Pinnacle and Acuros in Eclipse).

Fixed-field IMRT was adopted for the MR-Linac plans, with nine equally-spaced co-planar beams, starting with a gantry angle of 0^o^. Due to limited space within the MR-Linac bore, the VMAT isocenter could not always be replicated. For these cases, isocenter was as close to the middle of the treated vertebral body as possible. Plans were calculated using 1mm resolution, similar to conventional linac plans, using the ViewRay TPS advanced implementation (enabling dose volume histogram (DVH)-based planning constraints) and Monte Carlo dose computation algorithm (1% dose uncertainty).

Plan evaluation

Dosimetric endpoints comparing MR-Linac and conventional linac plans included both PTV and OAR considerations. For the PTV, these were target coverage (V_100%_, V_95%_, and V_90%_), maximum dose (D_max_) for heterogeneity, and conformity index (CI = V_Rx_/V_PTV_, where V_Rx_ is the prescription isodose volume and V_PTV_ is PTV volume). For the OARs/PRVs, these consisted of D_max_ and organ specific dose-volume thresholds (e.g., spinal cord V_12.3Gy_ and V_18Gy_, esophagus V_17.7Gy_, bowel V_17.4Gy_, liver V_30Gy_, and lung V_20Gy_). TPS calculated beam-on time was also evaluated. Statistical significance was determined using a two-tailed paired-samples t-test.

Phantom measurements

To evaluate deliverability/accuracy of the plans and TPS calculated dose distributions, various dosimetric measurements were made for two of the eight cases, representing two ends of the spectrum of PTV geometric complexity. These cases were #7, with the PTV solely including the vertebral body (i.e., a simple target) and #8 with the PTV fully wrapped around the spinal cord (i.e., the most challenging target). Dosimeters were radiochromic film, a commercial quality assurance (QA) diode array, and an ionization chamber.

EBT3 Gafchromic film (Ashland Specialty Ingredients, NJ) provided a high-resolution comparison between planned and delivered dose. The accuracy of film for measurements in relatively high magnetic field environments has been established [18-19]. Film sheets (10×10 cm^2^) were placed at the center of a 30×30×30cm^3^ water-equivalent plastic phantom (Solid Water, Gammex, Middleton, WI). Film was exposed when oriented in axial, sagittal, and coronal planes. To avoid saturation, QA plans were scaled so that maximum dose was <1000 cGy. Treatment fields were re-cast onto a CT of this phantom and the calculated planar dose corresponding to film location/orientation was used for comparison. The RIT software package (Radiological Imaging Technology, Colorado Springs, CO) was used for dosimetric comparison via global gamma analysis with 3%/3mm and 2%/2mm criteria (10% low-dose threshold). Planned dose distribution was resampled to the higher film resolution (scanned using red channel, 150 dots per inch (DPI)).

The plans were also measured using the commercially-available, MR-compatible ArcCHECK phantom (Sun Nuclear Corporation, Melbourne, FL) with an Accredited Dosimetry Calibration Laboratory (ADCL) calibrated ionization chamber (Exradin A18MRI, Standard Imaging, Middleton, WI, collecting volume 0.123 cc) placed in the central insert. The ArcCHECK (consisting of a helical diode array) with ion chamber represents a common approach to routine patient-specific QA with the MR-Linac [19-20]. Previous studies have demonstrated accuracy of using this ion chamber in the presence of the 0.35 T magnetic field [19-21]. Treatment fields were re-cast onto the cylindrical ArcCHECK phantom in the TPS and the SunNuclear QA software used the resulting RTdose DICOM file to extract the planned dose plane corresponding to the cylindrical surface within the phantom containing the diode array. Comparison between planned and measured dose surfaces was performed using the same global gamma criteria (3%/3mm and 2%/2mm criteria, with a 10% low-dose threshold).

## Results

Planning comparison

**Figure 2:**
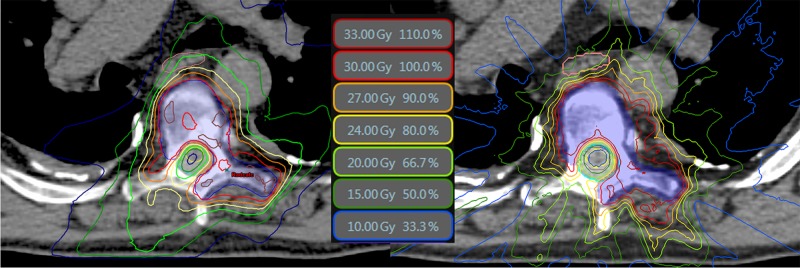
Qualitative Dosimetric Comparison Qualitative dosimetric comparison of isodose distribution for case #5 clinical volumetric modulated arc therapy (VMAT) plan (left) and corresponding magnetic resonance guided linac (MR-Linac) plan (right). The planning target volume (PTV) is the blue shaded region.

A representative case comparing the clinical VMAT and MR-linac plans is in Figure 2. The MR-linac plans spare the cord well but are less conformal (see isodose lines bowing out compared to VMAT plan, particularly near PTV distal to cord). The fixed-field IMRT approach for MR-linac planning exhibits more appreciable low-dose along beam directions. 

**Figure 3:**
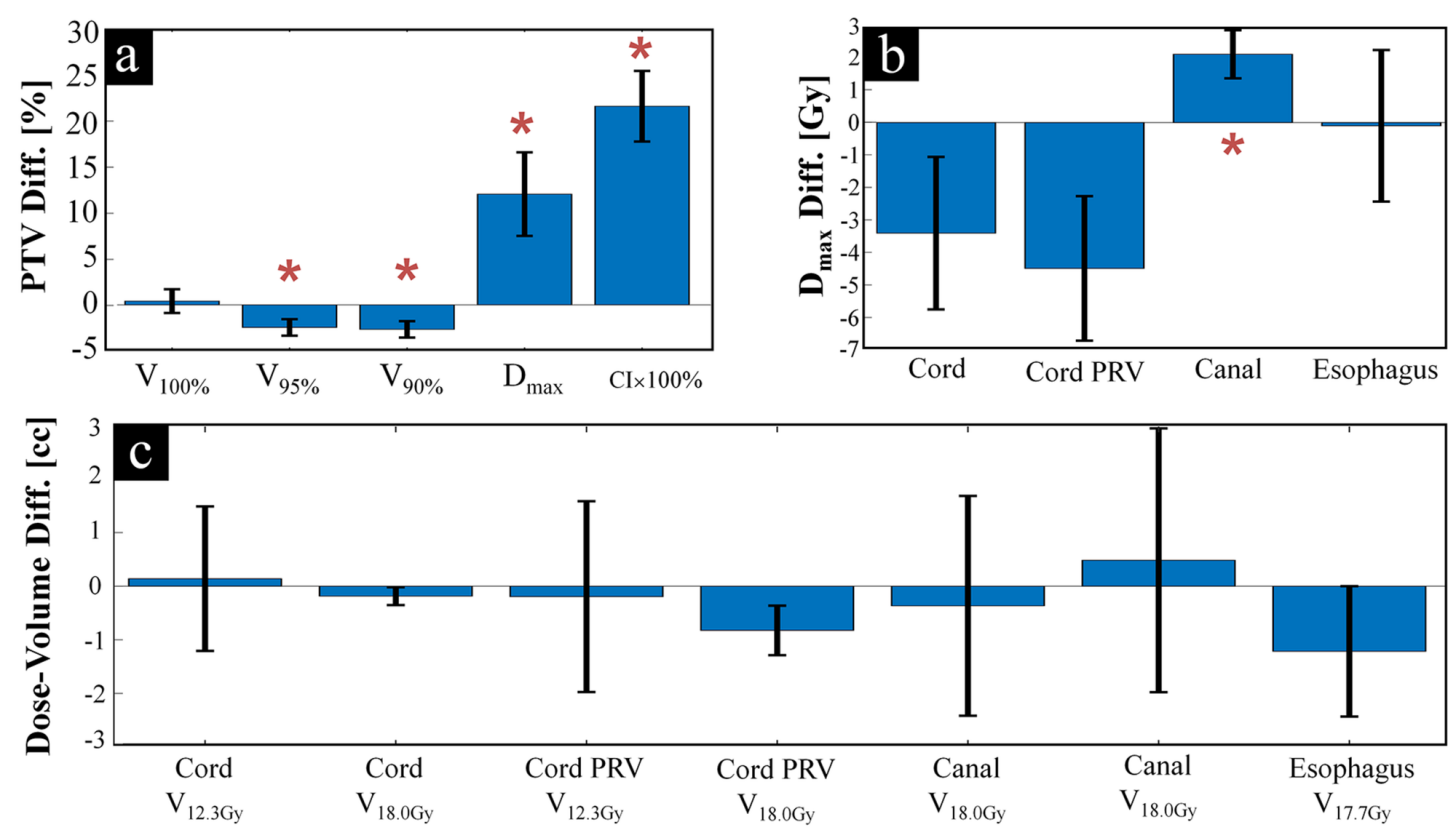
Quantitative Dosimetric Comparison Quantitative dosimetric comparison of planning target volume (PTV) parameters displayed in (a), Organ-at-risk/Planning-organ-at-risk-volume (OAR/PRV) max dose parameters are displayed in (b), and OAR/PRV dose-volume parameters are displayed in (c). Asterisks indicate statistically significant (p<0.05) differences. Differences are magnetic resonance guided-linac (MR-Linac) minus volumetric modulated arc therapy (VMAT) values. Error bars show standard deviation of this difference.

A comprehensive quantitative comparison of overall trends for all cases considered is in Figure 3. All MR-linac plans are clinically acceptable by our institutional spine SBRT protocol. There are no significant differences in target coverage (average PTV V_100_ of MR-linac vs. clinical VMAT are 92.7% [range: 85.9-95.9%] vs. 92.3% [range: 81.2-98.95%], respectively, p=0.7572). The higher conformity index demonstrates significantly inferior conformity for the MR-linac vs. VMAT plans (1.28±0.06 vs. 1.06±0.06, respectively, p=0.0005). Increased D_max_­ shows significantly increased heterogeneity for MR-linac vs. VMAT plans (134±3% vs. 120±2%, respectively, p=0.0270). While not statistically significant, MR-linac plans show trends of lower cord doses compared to VMAT plans (cord D_max_ of 16.1±2.7 Gy vs. 19.5±1.6 Gy, p=0.2066, and cord PRV D_max_ of 20.0±2.5 Gy vs. 24.5±2.0Gy, p=0.0996, for MR-linac vs. VMAT). Volumetric planning goals for the cord, cord PRV, spinal canal, and esophagus were comparable with no significant differences.

Plan deliverability

The planning comparison in Figure 2 and Figure 3 demonstrate a theoretical capability of the ViewRay MR-Linac to produce spine SBRT plans meeting clinical goals for various targets. To validate true deliverability, phantom measurements with various dosimeters assessed fidelity to planned dose distributions.

One aspect of deliverability is treatment time. Planning strategies avoiding excessive delivery times on the MR-linac are necessary. The number of segments during optimization was restricted and the IMRT “efficiency” parameter within the TPS was increased to encourage larger beam segments. The MR-linac plans consisted of the same nine gantry angles (40^o^ separation starting at 0^o^). The number of segments per IMRT beam were 9.21±0.48 (range: 2-21). The planning system calculated beam on time was 12.75±1.27 minutes (range: 8.85-17.92 minutes). Including gantry and MLC motion, the MR-linac treatment times were 14.39±1.26 minutes (range: 10.08-18.96 minutes), compared to the clinical VMAT treatment times of 9.57±1.19 minutes (p=0.0202). The MR-linac treatment times are significantly longer, but are still clinically reasonable.

Table 2 summarizes the phantom measurement results for cases #7 and #8 (representing the spectrum of potential PTV geometries, see Table 1 and Figure 1). Gamma passing rates for the diode array phantom and the film measurements are excellent with 3%/3mm and acceptable with 2%/2mm. Ion chamber measurements at a single point shows clinically acceptable fidelity (< 3% difference).

**Table 2:** Phantom Measurement Results Summary of results comparing measured to planned doses for cases #7 and #8. Gamma passing rates for both ArcCHECK diode and radiochromic film measurements using various gamma criteria are shown as well as a relative dose difference between ion chamber measured and planned dose.

	ArcCHECK		Film		Ion Chamber (Dose Difference)
Gamma Criteria:	3%/3mm	2%/2mm	3%/3mm	2%/2mm	
Case #7	100.0%	99.3%	98.0%	92.7%	+ 0.7%
Case #8	99.4%	95.3%	96.4%	89.3%	+ 2.5%

**Figure 4:**
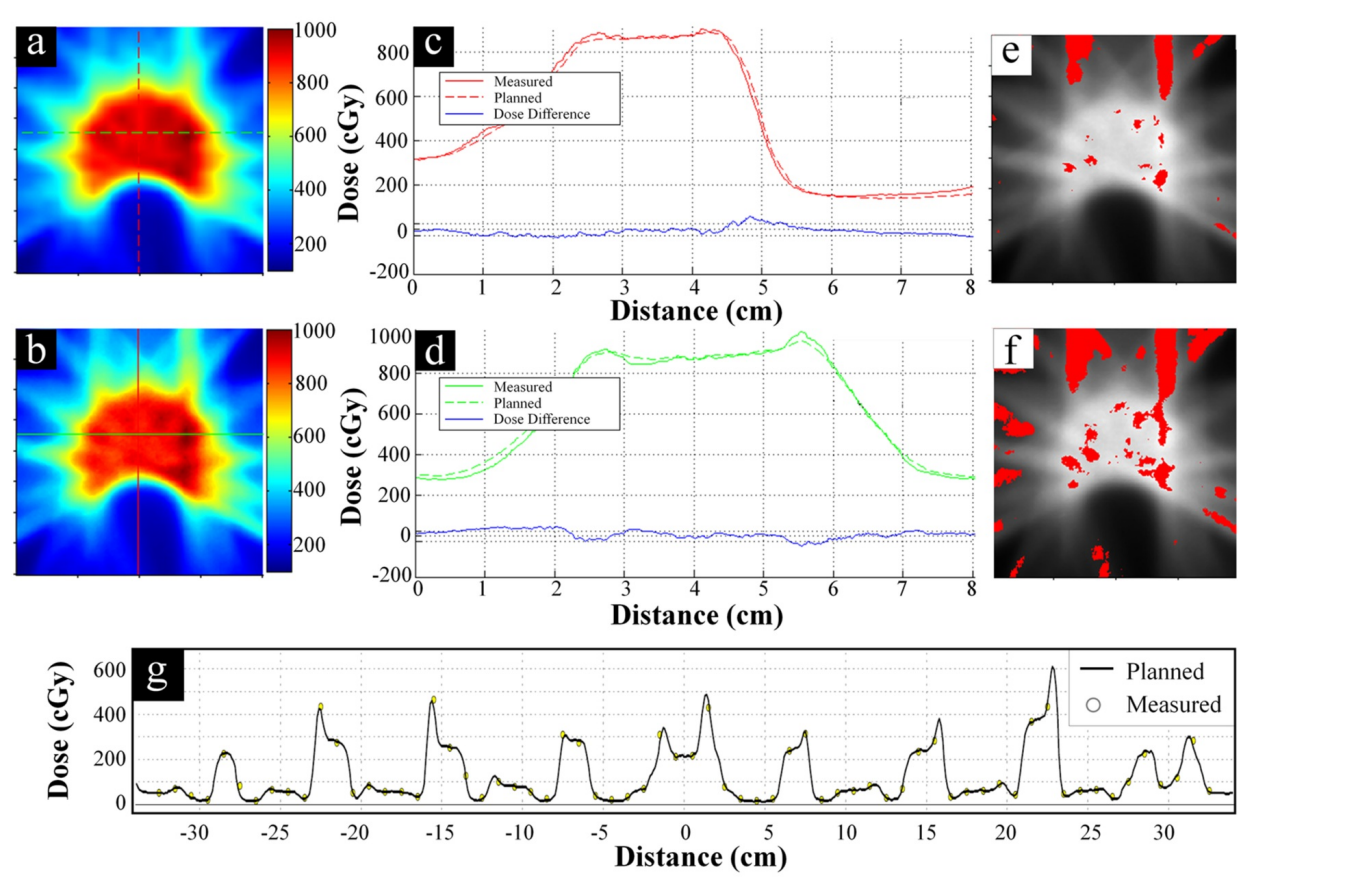
Case #7 - Simple PTV Geometry Spatial dosimetry results for case #7 representing simple planning target volume (PTV) geometry. (a) Planned and (b) measured planar dose distributions. (c) Vertical and (d) horizontal dose profiles along the red and green lines, respectively, shown in (a) and (b). Solid lines are measured profiles, dotted lines are planned profiles, and the blue line in (c) and (d) shows the difference between the two. Gamma analysis results when using either 3%/3mm or 2%/2mm criteria are shown in (e) and (f), respectively. Measured dose distribution is shown in grayscale and red points indicate a gamma value > 1 (i.e., failing points). (g) Representative dose profiles showing ArcCHECK diode measured (circles) versus planned (solid line) dose distributions. Yellow filled circles pass gamma analysis and blue/red filled circles fail with lower/higher measured dose.

**Figure 5:**
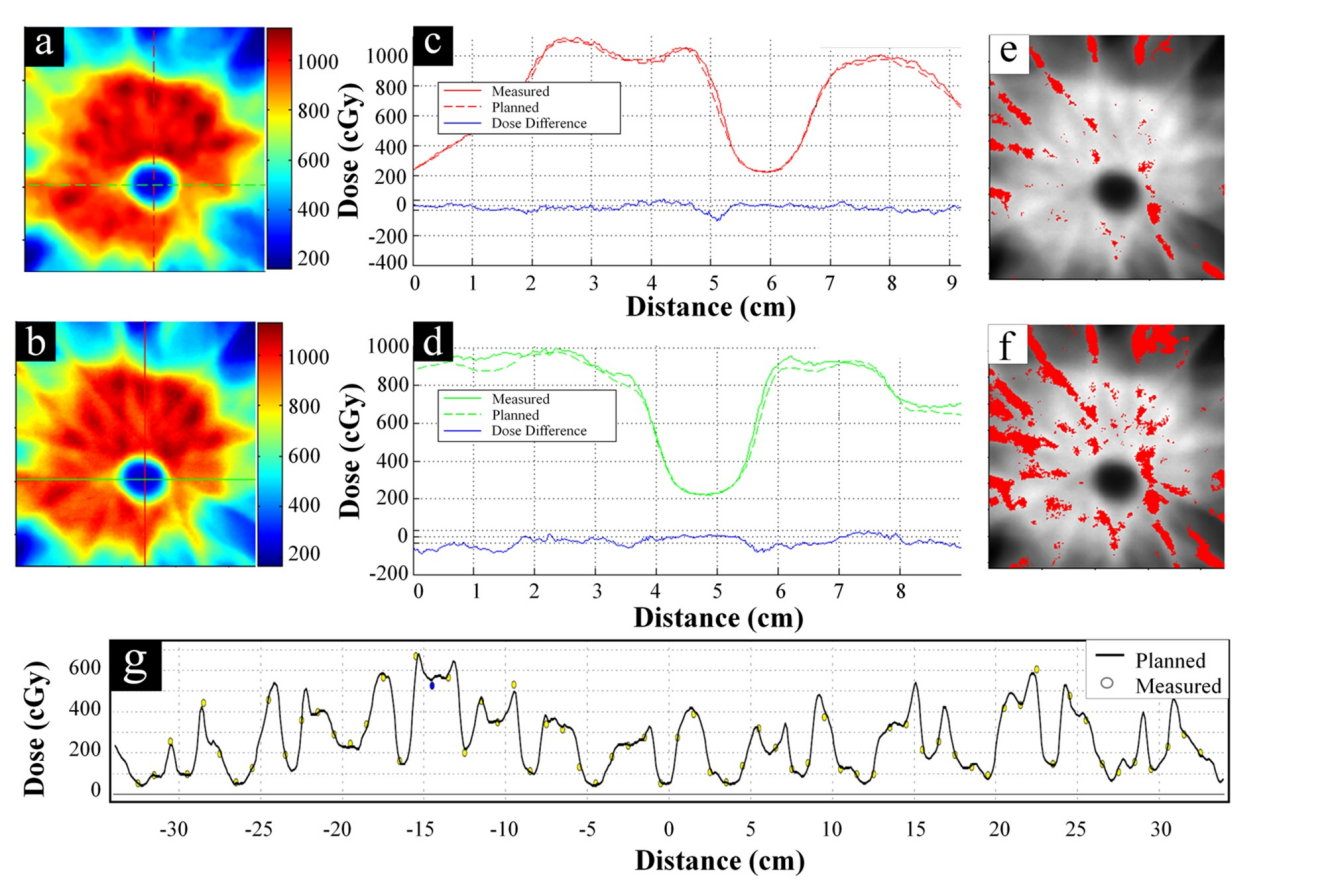
Case #8 - Complex PTV Geometry Spatial dosimetry results for case #8 representing a complex planning target volume (PTV) geometry. (a) Planned and (b) measured planar dose distributions. (c) Vertical and (d) horizontal dose profiles along the red and green lines, respectively, shown in (a) and (b). Solid lines are measured profiles, dotted lines are planned profiles, and the blue line in (c) and (d) shows the difference between the two. Gamma analysis results when using either 3%/3mm or 2%/2mm criteria are shown in (e) and (f), respectively. Measured dose distribution is shown in grayscale and red points indicate a gamma value > 1 (i.e., failing points). (g) Representative dose profiles showing ArcCHECK diode measured (circles) versus planned (solid line) dose distributions. Yellow filled circles pass gamma analysis and blue/red filled circles fail with lower/higher measured dose.

Film analysis is shown in Figure 4 and Figure 5 for cases #7 and #8, respectively. In Figure 5, the low-dose region for spinal cord sparing can be seen inside of the high-dose region used to treat the disease. Orthogonal dose profiles in Figure 4 and Figure 5 show closely matching spatial doses, which is particularly impressive in the high-gradient regions between the target and spinal cord. Regions failing gamma analysis for 3%/3mm and 2%/2mm criteria are shown in red in Figures 4e-4f and Figures 5e-5f. Figure 4g and Figure 5g shows similar dose profiles from diode array measurements.

## Discussion

Recent work found the MR-linac to be capable of cranial stereotactic radiosurgery (SRS) [18]. Extracranial or spinal SRS/SBRT utilizes similar margins (~1 mm) and requires similar precision/accuracy. This is facilitated by improvements between the ^60^Co and MR-linac iterations including: 6MV flattening filter free (FFF) beam (decreased penumbra, lower surface dose, higher penetration, and non-decaying 600 cGy/min dose rate), a 138 leaf double-stacked double-focused MLC system (effectively projecting to 0.415 cm rather than 1.05 cm at isocenter, enabling 0.2 cm x 0.4 cm field sizes and decreasing interleaf leakage) [15,18]. The relatively low magnetic field (0.35 T) decreases distortion and provides imaging accuracy better than 1 mm with registration accuracy on the order of a single voxel (~1.5 mm) [22].

Preliminary studies show that the ViewRay MRIdian linac can visualize the cord/cauda versus surrounding thecal sac [16]. While spine SBRT patients have been successfully and safely treated with current non-MRgRT approaches, most rely on bony anatomy as a surrogate for the true OAR (cord). PRV expansion partly accounts for cord motion independent of surrounding vertebra. However, registering images capturing intrafraction motion to initial planning images based on bony anatomy or the cord itself resulted in average setup differences of 0.9 mm (up to 1.8 mm) [16]. This suggests that we can improve spine SBRT by registering directly to the spinal cord. With no clear time trends to such motion, continuous/real-time image guidance, as provided by MR-linac may be necessary [16]. A system that can visualize the dose-limiting cord in real-time potentially enables cord-based gating. However, this may lengthen treatment times.

Different general approaches to online adaptive RT are discussed in the literature [7,23-25]. The work presented herein argues for spine SBRT implementation on the MRIdian MR-linac platform, but the proper adaptive approaches must be determined. Online plan reoptimization for spine SBRT must be investigated as has been done for other sites [24-26]. More accurate image guidance, gating, and/or adaptive RT for spine SBRT on the MR-linac may enable reduced margins and dose escalation for improved efficacy.

Compared to conventional linac VMAT plans, increased heterogeneity (greater D_^max^_) and inferior conformality (particularly for intermediate/lower isodoses) found in this work are consistent with previous studies [12]. It is important to emphasize that the heterogeneity, while higher for the MR-linac plans, was found to be clinically acceptable for treatments of this nature, as it facilitates the necessary steep dose fall off to spare the nearby spinal cord. For this work, nine equally-spaced, fixed IMRT fields were used. Prior studies found no significant improvements with greater than nine beams [27]. While presented results show dosimetric capabilities needed for spine SBRT, approaches may be improved (e.g., plan specific beam angles, off-axis treatments approximating non-coplanar beams, alternative optimization approaches, additional delivery efficiency considerations, etc.). Clinical VMAT plans had more freedom to utilize variable numbers of arcs (2-4), collimator angles, etc., based on target geometry and relationship to nearby OARs. Target and OAR contours were kept consistent between clinical VMAT and MR-linac plans. Other planning studies have incorporated assumed benefits of MRgRT on PTV/PRV margins and utilized smaller targets in the MRgRT plans, providing a dosimetric advantage [11,13-14]. In fact, the previous study exploring spine SBRT for the Cobalt system used this approach (VMAT: 1 mm cord PRV vs. ViewRay: cord PRV = true cord). Regardless, while the cord was well-visualized, dosimetry was inadequate [13]. The single fraction prescription of 18 Gy was decreased to 12.6 Gy on average to respect cord tolerance (versus 17.5 Gy for VMAT, which used an additional 1 mm margin for cord PRV). Average beam-on time (48.82 minutes) was clinically unacceptable [13]. With the MR-linac, target coverage is comparable to VMAT and clinically acceptable in all cases. While beam on time is longer than for VMAT, beam-on times with the 6MV FFF beam are acceptable (12.75±1.27 minutes), and related intrafraction motion issues arising from longer treatment times could potentially be offset by leveraging the OAR-based real-time tracking capabilities of the MR-Linac. Note, conventional linac plans used a 6MV beam (max dose rate of 600 MU/min), but if 6MV FFF (max dose rate of 1400 MU/min) were utilized, average treatment time (9.57±1.19 min) would decrease. It should be noted that the treatment planner was different for the MR-Linac plans and the clinical VMAT plans and that the MR-Linac planner was not necessarily blinded to the dosimetry of the baseline clinical VMAT plans.

Many spine SBRT patients require orthopedic hardware for spinal stabilization. While MR-compatible, it is unclear how susceptibility artifacts may affect MRgRT spine SBRT. However, a study investigating the MRIdian for high-dose rate (HDR) brachytherapy planning found decreased artifacts from applicators/instruments in 0.35 T versus higher-field diagnostic MRI [28]. MRI provides both functional and anatomical information. Diffusion-weighted imaging has already been demonstrated on the MRIdian [29]. As pulse-sequence options continue to expand, so will interesting clinical applications.

## Conclusions

This work demonstrates the feasibility of spine SBRT with fixed-field IMRT on the ViewRay MR-linac. Compared to clinical VMAT plans on a conventional linac, MR-linac plans had increased heterogeneity, lower conformality and longer beam-on. However, MR-linac plans were dosimetrically clinically acceptable and, while not statistically significant, tended to have improved cord sparing. Phantom measurements of delivered doses with an ion chamber, film, and diode array show good fidelity with planned doses, particularly in the regions between target and cord with large dose gradients, ensuring that the dose distributions are deliverable. MRgRT Spine SBRT is an application that may allow direct visualization of the dose-limiting spinal cord in real-time during treatment for reduced margins, dose escalation, gating, and/or adaptive radiotherapy.
